# Baseline metabolic profiles of early rheumatoid arthritis patients achieving sustained drug-free remission after initiating treat-to-target tocilizumab, methotrexate, or the combination: insights from systems biology

**DOI:** 10.1186/s13075-018-1729-2

**Published:** 2018-10-15

**Authors:** Xavier M Teitsma, Wei Yang, Johannes W G Jacobs, Attila Pethö-Schramm, Michelle E A Borm, Amy C Harms, Thomas Hankemeier, Jacob M van Laar, Johannes W J Bijlsma, Floris P J G Lafeber

**Affiliations:** 1 Department of Rheumatology & Clinical Immunology, University Medical Center Utrecht, Heidelberglaan 100, 3584 CX Utrecht, Netherlands; 2 Leiden Academic Center for Drug Research, Leiden University, 2300 RA Leiden, Netherlands; 3 Netherlands Metabolomic Centre, Einsteinweg 55, 2333 CC Leiden, Netherlands; 4 F. Hoffmann-La Roche, Grenzacherstrasse 124, 4070 CH Basel, Switzerland; 5 Roche Nederland BV, Beneluxlaan 2a, 3446 GR Woerden, Netherlands

**Keywords:** Rheumatoid arthritis, Tocilizumab, Methotrexate, Drug-free remission, Metabolomics

## Abstract

**Background:**

We previously identified, in newly diagnosed rheumatoid arthritis (RA) patients, networks of co-expressed genes and proteomic biomarkers associated with achieving sustained drug-free remission (sDFR) after treatment with tocilizumab- or methotrexate-based strategies. The aim of this study was to identify, within the same patients, metabolic pathways important for achieving sDFR and to subsequently study the complex interactions between different components of the biological system and how these interactions might affect the therapeutic response in early RA.

**Methods:**

Serum samples were analyzed of 60 patients who participated in the U-Act-Early trial (ClinicalTrials.gov number NCT01034137) and initiated treatment with methotrexate, tocilizumab, or the combination and who were thereafter able to achieve sDFR (*n* = 37); as controls, patients were selected who never achieved a drug-free status (*n* = 23). Metabolomic measurements were performed using mass spectrometry on oxidative stress, amine, and oxylipin platforms covering various compounds. Partial least square discriminant analyses (PLSDA) were performed to identify, per strategy arm, relevant metabolites of which the biological pathways were studied. In addition, integrative analyses were performed correlating the previously identified transcripts and proteins with the relevant metabolites.

**Results:**

In the tocilizumab plus methotrexate, tocilizumab, and methotrexate strategy, respectively, 19, 13, and 12 relevant metabolites were found, which were subsequently used for pathway analyses. The most significant pathway in the tocilizumab plus methotrexate strategy was “histidine metabolism” (*p* < 0.001); in the tocilizumab strategy it was “arachidonic acid metabolism” (*p* = 0.018); and in the methotrexate strategy it was “arginine and proline metabolism” (*p* = 0.022). These pathways have treatment-specific drug interactions with metabolites affecting either the signaling of interleukin-6, which is inhibited by tocilizumab, or affecting protein synthesis from amino acids, which is inhibited by methotrexate.

**Conclusion:**

In early RA patients treated-to-target with a tocilizumab- or methotrexate-based strategy, several metabolites were found to be associated with achieving sDFR. In line with our previous observations, by analyzing relevant transcripts and proteins within the same patients, the metabolic profiles were found to be different between the strategy arms. Our metabolic analysis further supports the hypothesis that achieving sDFR is not only dependent on predisposing biomarkers, but also on the specific treatment that has been initiated.

**Trial registration:**

ClinicalTrials.gov, NCT01034137. Registered on January 2010

**Electronic supplementary material:**

The online version of this article (10.1186/s13075-018-1729-2) contains supplementary material, which is available to authorized users.

## Background

Rheumatoid arthritis (RA) is a systemic disease characterized by inflammation and damage of the affected joints; although the cause is not known, both genetic as well as environmental factors are reported to be associated with the condition [1–4]. Initiating treatment, aiming for sustained remission or low disease activity early in the course of the disease, is important to preserve physical function and improve long-term prognosis [5–7]. Biological disease-modifying anti-rheumatic drugs (DMARDs) are to date mainly used as the second line of therapy in the management of early RA, although several studies showed their superior efficacy over traditional DMARDs in reducing disease activity and halting joint damage [8, 9]. Starting biological therapy in newly diagnosed RA patients as standard care, however, still remains highly controversial considering costs and unnecessary exposure to adverse events as a proportion of patients will be over-treated when using such an approach. Therefore, predictors are not only needed for treatment response to the currently recommended conventional DMARDs, but also for identifying patients for whom it would be favorable to initiate, as first therapy, a step-down biological-based strategy (i.e. tapering and finally discontinuing treatment) as achieving remission in the early stage of the disease improves the long-term clinical outcome.

Recent developments in “omics” technologies—such as genomics, transcriptomics, proteomics, and metabolomics—made it feasible to measure a broader spectrum of disease biomarkers for prediction of disease progression and development of personalized treatment strategies in RA [10]. Metabolomics is the non-targeted study of small-molecule metabolites and has become of increased interest in recent years due to the development and accessibility of new high-throughput technologies, including nuclear magnetic resonance spectroscopy and mass spectrometry (MS) [11]. Metabolites provide, under a given set of conditions, detailed information on cellular processes that are indicative for the disease state and are considered as the final downstream product of gene expression [12]. Especially in RA, metabolites are of particular interest as widespread cytokine-mediated inflammatory processes alter the cellular metabolism, when macrophages and lymphocytes become activated [13]. The role of these compounds in biomarker discovery has also been demonstrated previously, suggesting that metabolic analysis is potentially valuable in identifying markers for treatment response in patients with RA [14–17].

The aim of this study was to identify relevant metabolites and important metabolomic pathways associated with achieving sustained drug-free remission (sDFR) after a treat-to-target tocilizumab- or methotrexate-based strategy initiated in DMARD-naïve early RA patients. We previously identified, within the same patients, networks of co-expressed genes [18] and several inflammatory proteins [19] associated with sDFR and now, in the present study, by revealing metabolic biomarkers, exploring the systems biology of these patients in more detail by also integrating the findings of our previous studies.

## Methods

### Patient selection

From the U-Act-Early strategy trial, patients were selected who achieved sDFR, defined as being drug-free for ≥ 3 months until end of the study, after initiating tocilizumab, step-up methotrexate, or tocilizumab plus methotrexate therapy. As controls, we selected patients who never achieved a drug-free status at any time point during the study. A detailed description of the study design has been reported previously [20]. Briefly, DMARD-naïve patients with early RA were randomized (1:1:1) to one of the three strategy arms and treated to the target of sustained remission, defined as disease activity score assessing 28 joints (DAS28) < 2.6 with ≤ 4 swollen joints for ≥ 24 weeks. Tocilizumab was administered intravenously every four weeks at a dose of 8 mg/kg with a maximum of 800 mg. Methotrexate (oral) was given every week with a starting dose of 10 mg and was increased to 30 mg (or maximum tolerable dose) with steps of 5 mg every four weeks until remission was reached. If the treatment target was achieved, medication was tapered stepwise and finally discontinued, if remission persisted.

### Metabolomic platforms

Baseline serum samples were measured on oxidative stress, amines, and oxylipins MS platforms, which have been applied previously and are validated [21–23]. The oxidative stress platform covers various isoprostane classes, signaling lipids from the sphingosine and sphinganine classes and their phosphorylated forms, as well as three classes of lysophosphatidic acids: lysophosphatidic acids, alkyl-lysophosphatidic acids, and cyclic-lysophosphatidic acids (all in the chain length species range of C14–C22). The amine platform covers amino acids and biogenic amines and the oxylipin platform covers classical and non-classical eicosanoids from different polyunsaturated fatty acids. In total, 263 metabolites were measured for each sample on the different platforms: 57 signaling lipid mediators, 128 oxylipins, and 78 amines. Serum samples were thawed on ice and vortexed before preparation procedures following inner standard protocols [22, 23]; extra samples were pooled for internal quality control (QC). For each platform, QC samples were added and the relative standard deviation (RSD) per metabolite was calculated. Only those metabolites that complied with the acceptance criteria (RSD < 15% for amines; RSD < 30% for oxidative stress and oxylipins) were selected for further data analysis. Additional information about the metabolite profiling on the different platforms is provided in Additional file 1. All metabolite analyses were performed by the Biomedical Metabolomics Facility Leiden department at Leiden University.

### Data pre-processing

For peak determination and integration, signaling lipid mediators profiled by the oxidative stress platform were pre-processed by LabSolutions (Shimadzu, Version 5.65); peak-picking of oxylipins was performed with Agilent MassHunter Quantitative Analysis software (Agilent, Version B.05.01) and amines with MultiQuant Software for Quantitative Analysis (AB SCIEX, Version 3.0.2). For all metabolites, raw data correction was accomplished using selected internal standards by calculating the ratio of peak area of the target compound to the peak area of assigned internal standard from which a response ratio for each analyte was obtained. QC samples were used for evaluating the quality of the targeted compounds according to the in-house written protocol and the data were hereafter ready to be used for statistical analyses.

### Statistical analyses

Baseline clinical characteristics are described as mean (standard deviation [SD]), median (interquartile range [IQR]) or as proportions (%); between-group differences (sDFR versus controls) were tested within each strategy arm using independent *t* test, Mann–Whitney U test, or Pearson χ^2^ test, respectively. A linear mixed model with a random intercept and baseline DAS28, week of visit, and group (sDFR versus controls) as fixed effects was built to evaluate, within the strategy arms, differences in disease activity over time. As metabolite concentrations are influenced by a variety of factors, we performed principal component analyses (PCA) to identify possible confounders. The following parameters were considered: age; body mass index, gender, ethnicity, disease duration, smoking, alcohol consumption, seropositivity for rheumatoid factor (RF) or cyclic citrullinated peptide (CCP), erythrocyte sedimentation rate (ESR), and C-reactive protein (CRP). Thereafter, supervised partial least square discriminant analyses (PLSDA) were performed for each class (lipids, amines, and oxylipins) to identify relevant metabolites within each strategy arm. Several multivariate discrimination techniques currently exist but the main advantage of PLSDA is the handling of collinearity and noisy data (i.e. more observations than samples), both common in metabolomics experiments [24]. Data were first normalized (natural log-transformed) and then standardized (*z*-score) to ensure that all metabolite scores are comparable by giving them equal weight. The variable importance on projection (VIP) was used for metabolite selection; this measure accumulates the importance of each variable, whereas a higher VIP score shows that it is more relevant to predict the outcome [25]. Metabolites with VIP > 1 in the first component were considered important as the squared sum of all VIP values is equal to “1”, i.e. the average VIP. Thereafter, the Mann–Whitney U test (sDFR versus controls) was performed within these selected metabolites to identify those who are most relevant (*p* < 0.10) within each strategy arm (not corrected for multiple testing), which were subsequently used for pathway analysis in the Kyoto Encyclopedia of Genes and Genomes (KEGG) databases. Pathways were considered relevant when *p* ≤ 0.05. In addition, integrative analyses between the previously identified transcripts [18] and proteins [19] and relevant metabolites were performed by calculating statistically significant (*p* ≤ 0.05) transcript–protein and protein–metabolite correlations (Pearson correlation coefficients [PCC]). These network analyses were visualized using VisANT 5.0 software [26, 27]. All other analyses were performed using the web-based tool MetaboAnalyst version 4.0 [28] and R version 3.4.3 (R Foundation for Statistical Computing, Vienna, Austria).

## Results

**Fig. 1:**
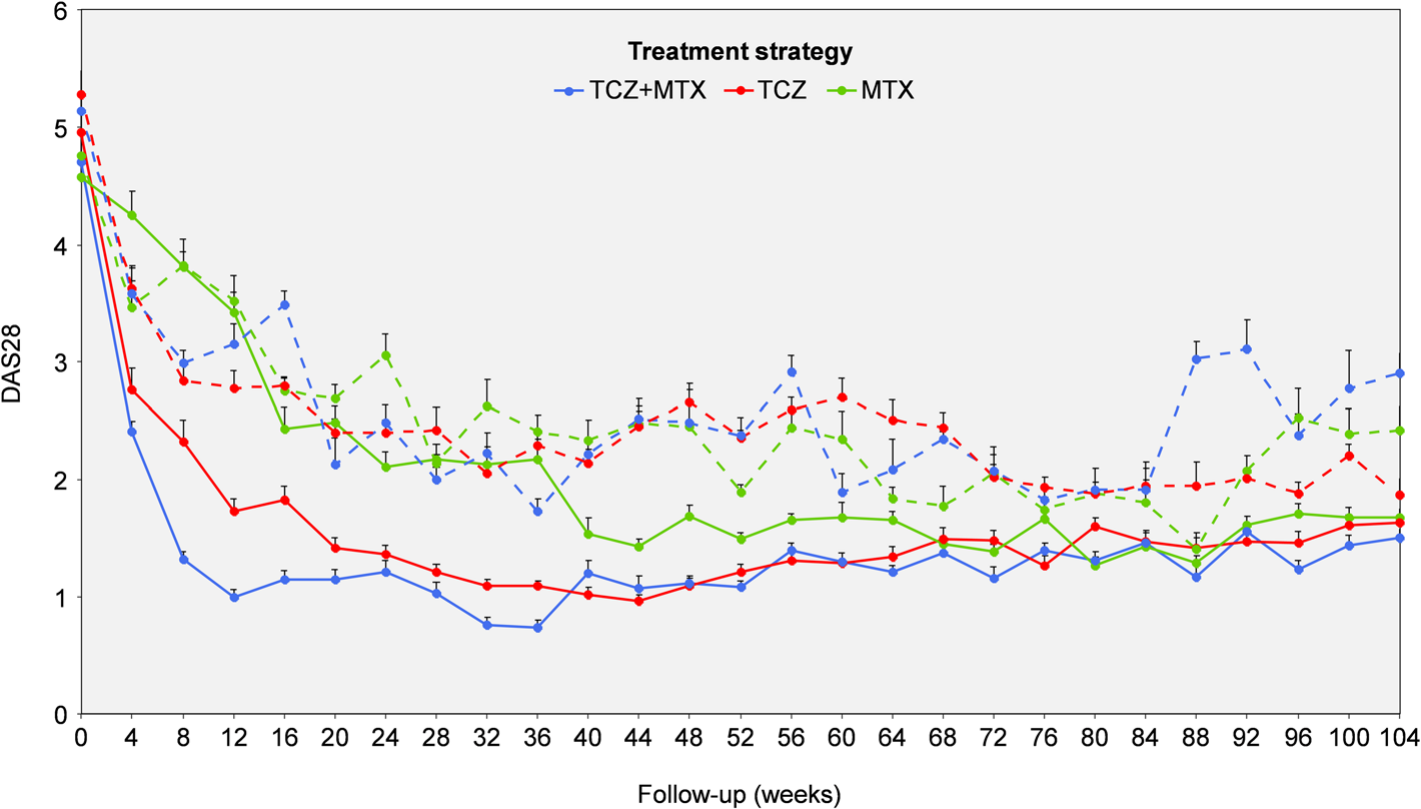
Mean (SE) DAS28-ESR over time in those achieving sDFR (*continuous line*) *vs* controls (*dotted line*) within the three strategy arms. DAS28 disease activity score assessing 28 joints, ESR erythrocyte sedimentation rate, MTX methotrexate, sDFR sustained drug-free remission, SE standard error, TCZ tocilizumab

Serum samples were analyzed for 60 patients (tocilizumab plus methotrexate arm: *n* = 14 sDFR, *n* = 5 controls; tocilizumab arm: *n* = 13 sDFR, *n* = 11 controls; methotrexate arm: *n* = 10 sDFR, *n* = 7 controls) and their baseline characteristics are summarized in Table 1. The mean (SD) age of all patients was 53 (14) years with a median (IQR) symptom duration of 23 (18–40) days; the majority was seropositive for rheumatoid factor (60%) or cyclic citrullinated peptide (60%). At baseline, the mean (SD) DAS28 of these patients was 4.9 (1.1) with a median (IQR) ESR of 20 (11–32) mm/1^th^h and median (IQR) CRP of 9 (3–18) mg/L. No significant differences were noted in clinical characteristics at baseline between the groups (sDFR versus controls) within the strategy arms (*p* ≥ 0.07). The mean (standard error) DAS28 scores over time of the sDFR and control groups are shown in Fig. 1. In the longitudinal analysis, significant lower DAS28 scores were found in the sDFR group within the tocilizumab plus methotrexate (mean – 1.18, 95% confidence interval [CI] – 0.87, – 1.50; *p* < 0.001), tocilizumab (mean – 0.87, 95% CI – 0.62, – 1.12; *p* < 0.001), and methotrexate (mean − 0.43, 95% CI -0.16, − 0.69; *p* = 0.009) arms, when compared to the control group.

**Table 1:** Baseline characteristics of the patients included in the analyses

	Tocilizumab plus methotrexate		Tocilizumab		Methotrexate	
	sDFR (*n* = 14)	Controls (*n* = 5)	sDFR (*n* = 13)	Controls (*n* = 11)	sDFR (*n* = 10)	Controls (*n* = 7)
Female gender, *n* (%)	6 (43)	4 (80)	9 (69)	8 (73)	8 (80)	6 (86)
Age (years)	53 (16)	64 (10)	58 (14)	51 (13)	50 (14)	46 (17)
BMI (kg/m^2^)	25 (4)	27 (4)	25 (2)	25 (5)	29 (4)	26 (3)
Caucasian, *n* (%)	13 (93)	4 (80)	13 (100)	10 (91)	10 (100)	7 (100)
Current smokers, *n* (%)	3 (21)	1 (20)	2 (15)	3 (27)	1 (10)	1 (14)
Symptom duration (days), median (IQR)	22 (21–40)	19 (14–55)	24 (18–39)	21 (16–25)	30 (13–40)	31 (20–45)
RF positive, *n* (%)	5 (34)	3 (60)	8 (62)	6 (55)	9 (90)	5 (71)
Anti-CCP positive, *n* (%)	5 (34)	3 (60)	8 (62)	7 (64)	7 (70)	6 (86)
CRP (mg/L), median (IQR)	5 (2–13)	5 (4–9)	15 (4–27)	14 (4–30)	11 (5–18)	5 (4–12)
ESR (mm/h), median (IQR)	18 (12–39)	25 (23–29)	26 (14–28)	20 (9–39)	25 (13–47)	16 (13–25)
DAS28 (range 0–9.4, 9.4 = maximum)	4.7 (1.2)	5.1 (0.9)	5.0 (1.1)	5.3 (1.3)	4.6 (1.2)	4.8 (0.9)
HAQ (range 0–3, 3 = worst function)	0.8 (0.5)	1.5 (0.9)	1.0 (0.6)	1.4 (0.7)	0.9 (0.6)	1.0 (0.5)
Sharp/van der Heijde score, median (IQR)	0 (0–0)	0 (0–0)	0 (0–3)	0 (0–2)	0 (0–1)	0 (0–0)

Continuous data presented as mean (SD) unless otherwise indicated

*SD* standard deviation, *IQR* interquartile range, *sDFR* sustained drug-free remission, *BMI* body mass index, *RF* rheumatoid factor, *CCP* cyclic citrullinated peptide, *CRP* C-reactive protein, *ESR* erythrocyte sedimentation rate, *DAS28* disease activity score assessing 28 joints, *HAQ* health assessment questionnaire

### Metabolite biomarkers for sDFR

PCA revealed no clear confounders (data not shown) and therefore metabolite concentrations were not corrected for clinical characteristics in further analyses. PLSDA identified 35 metabolites (15 signaling lipid mediators, 14 oxylipins, 6 amines) in the tocilizumab plus methotrexate arm, 33 metabolites (9 signaling lipid mediators, 15 oxylipins, 9 amines) in the tocilizumab arm, and 33 metabolites (11 signaling lipid mediators, 13 oxylipins, 9 amines) in the methotrexate arm. Of these metabolites, 19, 13, and 12, respectively, were subsequently selected for further pathway analyses (Table 2). When comparing the metabolites between the strategy arms, one (LPA c16:1) showed overlap in the tocilizumab plus methotrexate and tocilizumab arm; two (12,13-DiHODE and LPA c16:0) in the tocilizumab plus methotrexate and methotrexate strategy, and one (3-Methylhistidine) in the tocilizumab and methotrexate strategy.

**Table 2:** Identified metabolites associated with achieving sustained drug-free remission in the three strategy arms

Tocilizumab plus methotrexate	SMD	*p* value	Tocilizumab	SMD	*p* value	Methotrexate	SMD	*p* value
Histamine▼	− 1.75	0.002	PGE2▲	0.93	0.019	L-Lysine▲	0.95	0.032
9,12,13-TriHOME▲	0.52	0.002	L-Pipecolic acid▲	0.42	0.026	L-Proline▲	0.98	0.040
Spha c18:0▼	− 1.34	0.007	8,9-DiHETrE▲	0.81	0.026	3-Methylhistidine▼	− 1.05	0.06
9,10,13-TriHOME▲	1.05	0.010	5,6-DiHETrE▲	0.79	0.034	Anserine▲	0.73	0.06
LPA c20:3▼	− 1.29	0.012	8-iso-PGE2▲	0.89	0.034	19,20-DiHDPA▼	− 1.03	0.06
Sph c18:1▼	− 0.83	0.012	20-carboxy-LTB4▲	0.50	0.035	5-Hydroxy-L-tryptophan▼	− 0.40	0.08
LPA c18:1▼	− 0.93	0.033	Cystathionine▼	− 0.96	0.052	L-Arginine▲	0.98	0.08
L-Methionine sulfoxide▲	1.01	0.033	Norepinephrine▼	− 0.39	0.052	LPA c18:3 (w3/w6)▲	0.80	0.08
8,9-DiHETrE▼	− 0.94	0.042	3-Methylhistidine▼	− 0.61	0.07	12,13-DiHODE▼	− 0.60	0.10
LPA c16:0▼	− 0.81	0.052	TXB2▲	0.82	0.08	14,15-DiHETE▼	− 0.96	0.10
NO2-OA▼	− 0.98	0.052	8-iso-PGA2▲	0.58	0.08	cLPA c16:0▲	0.71	0.10
L-Kynurenine▼	− 1.03	0.06	aLPA c16:1▼	− 0.24	0.08	PAF c16:0▲	0.87	0.10
LPA c22:4▼	− 1.03	0.06	Homocysteine▼	− 0.78	0.09			
LPA c20:4▼	− 0.97	0.06						
Methyldopa▼	− 0.88	0.08						
PGD2▲	0.02	0.08						
LPA c16:1▼	− 0.84	0.08						
Hydroxylysine▲	0.66	0.10						
PGF3a▲	0.07	0.10						
12,13-DiHODE▲	0.85	0.10						

▲On average, higher concentration in the sDFR group *vs* controls; ▼on average, lower concentration in the sDFR group *vs* controls. *sDFR* sustained drug-free remission, *SDM* standardized mean difference

### Pathway analyses

**Fig. 2:**
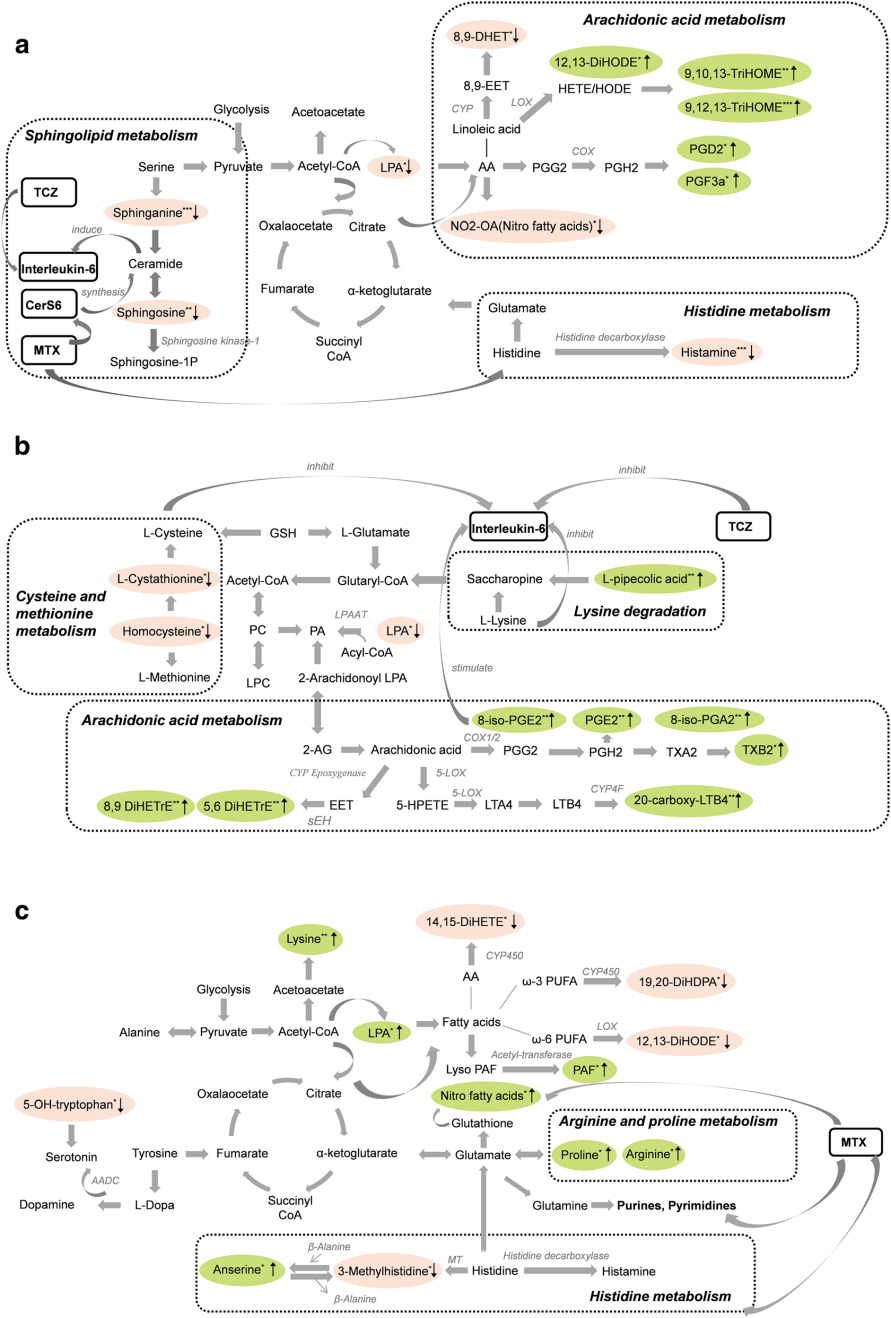
Pathway analysis within the identified metabolites in the (**a**) tocilizumab plus methotrexate, (**b**) tocilizumab, and (**c**) methotrexate strategy arms. Metabolites depicted in *red* nodes have, on average, lower concentration in the sDFR group compared to controls; those depicted in *green* nodes have a higher concentration. *p ≤ 0.10, **p ≤ 0.05, ***p ≤ 0.01. Metabolites not included in the top three most relevant pathways are not displayed

Pathway overviews for each strategy arm are shown in Additional file 2. The three most relevant KEGG pathways in the tocilizumab plus methotrexate arm were “histidine metabolism” (*p* < 0.001), “sphingolipid metabolism” (*p* = 0.004), and “arachidonic acid metabolism” (*p* = 0.037). Within the “histidine metabolism” pathway, a significant lower concentration of histamine (*p* = 0.002) was found in the sDFR group when compared to controls (Fig. 2). In the “arachidonic acid metabolism” pathway, production of PGD2 and 12,13-DiHODE, 9,10,13-TriHOME, and 9,12,13-TriHOME through lipoxygenase was higher in those who achieved sDFR. Within the “sphingolipid metabolism” pathway, significantly lower levels of sphinganine (Spha c18:0, *p* = 0.007) and sphingosine (Sph c18:1, *p* = 0.012) were found in the sDFR (versus controls) group, which are both related to ceramide generation. In the tocilizumab arm, three most relevant pathways were identified: “arachidonic acid metabolism” (*p* = 0.018); “lysine degradation” (*p* = 0.023); and “cysteine and methionine metabolism” (*p* = 0.030, Fig. 2). In the “arachidonic acid metabolism” pathway, higher concentrations of prostaglandin E2 (PGE2), 8-isoprostaglandin E2 (8-iso-PGE2), prostaglandin A2 (PGA2), 8-isoprostaglandin A2 (8-iso-PGA2), 8,9-DiHETrE, and 5,6-DiHETrE (*p* ≤ 0.08) were found in the sDFR group, compared to controls, implying a more active role of prostaglandins and isoprostanes, which are critical signaling molecules in various inflammatory disease including RA [29–31]. Furthermore, in the “lysine degradation” pathway, significantly higher concentrations were found in the sDFR group (versus controls) of L-pipecolic acid (*p* = 0.026) and in the “cysteine and methionine metabolism” pathway, slightly but not statistically significantly lower concentrations were found in the sDFR group for cystathionine (*p* = 0.052) and homocysteine (*p* = 0.09). Most metabolites in the methotrexate arm were associated with the “arginine and proline” and “histidine metabolism” pathways (*p* = 0.022 and *p* = 0.025, respectively). When compared to controls, lower oxylipin levels of 14,15-DiHETE, 19,20-DiHDPA, and 12,13-DiHODE were found in the sDFR group (*p* ≤ 0.10), which indicates fewer active cytochromes P450 (CYP450) and lipoxygenase-based fatty acids metabolism, while the observed increased L-proline (*p* = 0.040) and L-arginine (*p* = 0.08) levels suggest a more active role of this pathway (Fig. 2). In the “histidine metabolism” pathway, decreased levels of 3-methylhistidine (*p* = 0.06) and higher levels of anserine (*p* = 0.06) were observed in the sDFR group (versus controls). Other changes of amine levels included higher concentrations of lysine (*p* = 0.032) and lower concentrations of 5-hydroxy-tryptophan (*p* = 0.08). Another significant pathway in the methotrexate arm was “aminoacyl-tRNA biosynthesis” (*p* = 0.027).

### Systems biology: from transcripts to proteins and metabolites

**Fig. 3:**
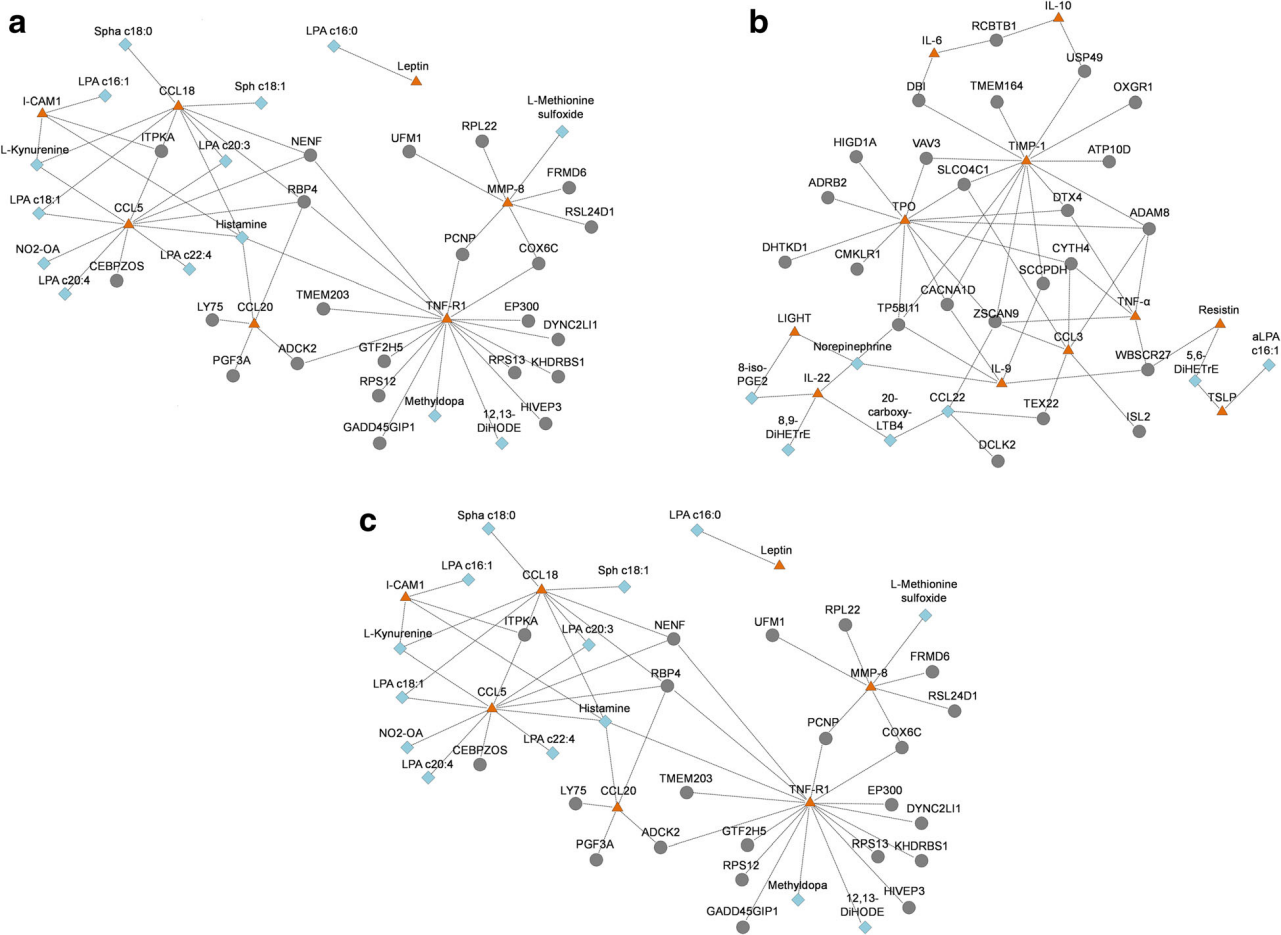
Network correlation between transcriptomic (*gray* nodes), proteomic (*orange* nodes), and metabolomic (*blue* nodes) biomarkers in the (**a**) tocilizumab plus methotrexate, (**b**) tocilizumab, and (**c**) methotrexate strategy arms. Only significant transcriptomic–proteomic and proteomic–metabolomic correlations are displayed

Figure 3 shows significant transcript–protein and protein–metabolite correlations within the three strategy arms. The average PCC between the biomarkers in the tocilizumab plus methotrexate arm was 0.54; these were 0.48 and 0.57 in the tocilizumab and methotrexate arm, respectively. Biomarkers showing > 10 connections within the networks were considered most important (i.e. signature biomarker) as they show the highest connectivity and therefore contribute most to the pathway analyses. The signature biomarkers in the tocilizumab plus methotrexate arm were the protein chemokine (C-C motif) ligand 5 (CCL5), as it was significantly correlated to seven metabolites and four transcripts, and the protein tumor necrosis factor receptor 1 (TNF-R1), being significantly correlated to three metabolites and 14 transcripts. In the tocilizumab arm, the proteins TIMP metallopeptidase inhibitor 1 (TIMP-1, 13 with transcripts) and thyroid peroxidase (TPO, 12 with transcripts) showed the highest number of correlations and in the methotrexate arm the protein granulocyte colony-stimulating factor (G-CSF, 4 with metabolites; 26 with transcripts).

## Discussion

We identified several small-molecule metabolites, by using high-throughput MS, associated with achieving sDFR after treatment with tocilizumab- or methotrexate-based strategies in newly diagnosed RA patients. In line with our previous observations, by measuring transcripts and proteins within the same patients, different metabolic profiles were found between the treatment strategies, further supporting the hypothesis that achieving sDFR is likely dependent on pre-treatment concentrations of specific biomarkers as no differences in clinical characteristics could be found. Although we did find different metabolic pathways between the treatment strategies when using the identified metabolites, the pathways within each strategy arm were found to be specific for the respective treatment, which shows the possibility of selecting biomarkers for prediction of a good treatment-specific response.

An important metabolic pathway within the tocilizumab plus methotrexate strategy was “sphingolipid metabolism,” in which ceramide synthases is closely associated with the consumption of both sphingosine, a lipid signaling molecule stimulating several cellular processes important in RA, such as cell growth, differentiation, and migration [32], and its derivative sphinganine [33]. These metabolites have been found to induce IL-6 production and may thus influence the treatment response to tocilizumab, a humanized monoclonal antibody against the IL-6 receptor. In the present analysis, lower baseline levels of sphingosine and sphinganine were found in the sDFR group of the tocilizumab plus methotrexate strategy arm when compared to controls although the opposite was expected (i.e. less inhibition resulting in higher disease activity); a possible explanation for these findings could be that patients with higher levels of IL-6 are more likely to respond to tocilizumab. To test this hypothesis, we evaluated within the same patients the absolute IL-6 concentrations in baseline (i.e. pre-treatment) serum, as was measured previously using Luminex® [19], but found no statistically significant difference between those who would later achieve sDFR and the controls (mean 65 versus 70 pg/mL, respectively). This result confirms the findings of others showing that baseline levels of IL-6 are not predictive of clinical outcomes of tocilizumab treatment [34]. Further studies are required to elucidate on how metabolite levels are altered and how these pathophysiological changes eventually affect the response to specific therapies. Ceramide synthase 6 (CerS6), an enzyme also important in sphingolipid biosynthesis, is mediated by methotrexate [35], which might indicate a role of sphingolipids in the treatment response to a tocilizumab plus methotrexate-based strategy in early RA.

In the tocilizumab strategy arm, several involved metabolic pathways were found to be associated with signaling of the IL-6 protein whereas increased levels of prostaglandins and isoprostanes were observed in the sDFR group when compared to controls. These metabolites are involved in the metabolism of arachidonic acid, a polyunsaturated fatty acid reported to be a key intermediate promoting inflammation. Prostaglandins are known to have a stimulating effect on IL-6 and could thus influence the response to tocilizumab therapy [36]. Other important metabolites involved in IL-6 signaling are L-pipecolic acid, which is produced during the degradation of lysine, and cysteine, which is synthesized from serine. Both lysine and serine are essential amino acids in humans as they are being used in the biosynthesis of proteins whereas lysine has been reported to downregulate the release of IL-6 [37].

In the methotrexate strategy, important metabolites were proline and arginine for which higher concentrations were observed in those achieving sDFR when compared to controls. Arginine, apart from its role in protein synthesis, serves as the precursor of proline and glutamate; glutamates are involved in the generation of glutamine, which stimulates purine and pyrimidine formation that is required for cell proliferation. These organic compounds thus seem to play a crucial role in the direct treatment response to methotrexate as this drug antagonizes folic acid [38], which is important for purine and pyrimidine formation, and inhibits via this pathway the synthesis of nucleic acids and subsequently protein synthesis [39]. One of the other important amino acids for protein synthesis is histidine, of which the post-translational modified product, 3-methylhistidine, was found to have a decreased metabolism in the present study in those achieving sDFR in the methotrexate strategy. Histidine is also involved in the local immune response as it is a precursor of histamine, a compound that induces permeability of capillaries allowing, for example, leukocytes and pro-inflammatory molecules to elicit an immune response. These amino acids thus might directly affect the treatment response to methotrexate therapy.

There are some limitations to this study. First, although not uncommon in metabolomic studies, the number of samples measured was relatively small, enhancing the likelihood of false-negative findings (i.e. type II error). To minimize this risk, we used for detecting relevant metabolites analyses suitable for handling such datasets consisting of more markers than samples. Second, before the samples were measured, serum was pre-processed according to usual guidelines, which differed for each platform, and compounds were, after analyses, corrected using internal standards. These variety of factors could potentially impair replicating or external validation of findings. Third, for defining remission, and thus tapering medication, we used DAS28 criteria which is highly dependent of acute phase response and might not always reflect an inflammation-free state of the patient. To minimize this risk, no more than four swollen joints were allowed during the remission period, enhancing the likelihood that patients were also clinically in remission. Nevertheless, American College of Rheumatology (ACR)/European League Against Rheumatism (EULAR) Boolean-based remission criteria might have been a more reliable tool for assessing disease activity as it is more stringent in assessing inflammation [40].

## Conclusions

We have identified several relevant metabolites in baseline serum related to achieving sDFR after treatment with tocilizumab- or methotrexate-based strategies in DMARD-naïve RA patients. In line with our previous work on the analyses of transcripts and proteins, performed within the same patients, the identified metabolic pathways were shown to be specific for the treatment that was initiated. These results might provide further insight into the role of predisposing biomarkers for eventually achieving sDFR in early RA. Signature metabolite biomarkers have been identified which could potentially serve as key prognostic factors for developing personalized care but need to be validated in large replication studies. Further studies are also warranted to elucidate on the drug metabolism in those patients with refractory disease to, by initiating other therapies based on pharmacogenomics, achieve better treatment outcomes.

## Additional files

Additional file 1: Additional information regarding the metabolite profiling on the three platforms. (DOCX 15 kb)

Additional file 2: Overview of the pathway analysis in the (a) tocilizumab plus methotrexate, (b) tocilizumab, and (c) methotrexate strategy arms. The top three most relevant pathways in the tocilizumab plus methotrexate arm were (1) “histidine metabolism,” (2) “sphingolipid metabolism,” and (3) “arachidonic acid metabolism;” in the tocilizumab arm, these were (1) “arachidonic acid metabolism,” (2) “lysine degradation,” and (3) “cysteine and methionine metabolism;” in the methotrexate arm, these were (1) “arginine and proline metabolism,” (2) “histidine metabolism,” and (3) “aminocyl-tRNA biosynthesis.” KEGG Kyoto Encyclopedia of Genes and Genomes, tRNA transfer ribonucleic acid. The colors of the nodes, varying from *yellow* to *red*, indicates the level of significance with *red* being highly significant; the size of the nodes depicts the impact of the pathway with larger nodes illustrating a higher impact. (DOCX 54 kb)

## Abbreviations

8-iso-PGE2: 8-isoprostaglandin E2
ACR: American College of Rheumatology
CCL5: Chemokine (c-c motif) ligand 5
CCP: Cyclic citrullinated peptide
CerS6: Ceramide synthase 6
CI: Confidence interval
CRP: C-reactive protein
CYP450: Cytochromes P450
DAS28: Disease activity score assessing 28 joints
DMARDs: Disease-modifying anti-rheumatic drugs
ESR: Erythrocyte sedimentation rate
EULAR: European League Against Rheumatism
G-CSF: Granulocyte colony-stimulating factor
IL: Interleukin
IQR: Interquartile range
KEGG: Kyoto Encyclopedia of Genes and Genomes
MS: Mass spectrometry
PCA: Principal component analyses
PGA2: Prostaglandin A2
PGE2: Prostaglandin E2
PLSDA: Partial least square discriminant analyses
QC: Quality control
RA: Rheumatoid arthritis
RF: Rheumatoid factor
SD: Standard deviation
sDFR: Sustained drug-free remission
TIMP1: TIMP metallopeptidase inhibitor 1
TNF-R1: Tumour necrosis factor receptor 1
TPO: Thyroid peroxidase
VIP: Variable importance on projection
