# Supplementary material for: Baseline metabolic profiles of early rheumatoid arthritis patients achieving sustained drug-free remission after initiating treat-to-target tocilizumab, methotrexate, or the combination: insights from systems biology
Source: Arthritis Res Ther. 2018 Oct 15;20:230. doi: 10.1186/s13075-018-1729-2 (PMC6235217; doi:10.1186/s13075-018-1729-2)
Supplement: Supplementary file 1 — Additional information regarding the metabolite profiling on the three platforms. (DOCX 15 kb) [file 13075_2018_1729_MOESM1_ESM.docx]

| **Additional file 1:** Additional information regarding the metabolite profiling on the three platforms | |
| --- | --- |
| *Oxidative stress profiling* | For measuring different classes of lipids in this platform, low pH and high pH chromatography was applied respectively. Isoprostanes, prostaglandins, nitro-fatty acids and lyso-sphingolipids were included in low pH application while high pH chromatography covered lyso-sphingolipids, lysophosphatidic acids, alkyl-lysophosphatidic acids and cyclic-phosphatidic acids. Liquid-liquid extraction (LLE) was employed to extract metabolites in each 150 μL serum aliquot spiked with internal standards (ISTDs) and citric acid/phosphate buffer (pH 4.5) followed by butanol and ethyl acetate extraction. The organic phase was collected and concentrated by drying and then reconstituted with a smaller volume. The re-constituent was divided into two vials and were injected with low pH and high pH chromatography respectively. Ultra-performance liquid chromatography tandem mass spectrometry (UPLC-MS/MS, Shimadzu LCMS-8050, Japan) with an electrospray ionisation (ESI) source was applied for sample analysis. |
| *Oxylipins profiling* | Oxylipins platform measures oxygenated metabolites derived from different polyunsaturated fatty acids (PUFAs) including linoleic acid and arachidonic acid (both n-6 PUFAs) and eicosapentaenoic acid (EPA) and docosahexaenoic acid (DHA) (both n-3 PUFAs). Serum samples aliquoted to 250 μL volume were spiked with antioxidant and ISTDs and were conducted with solid phase extraction applying a hydrophilic-lipophilic balance (HLB) SPE cartridge (Oasis, Waters). After being eluted with methanol and ethyl acetate, oxylipins were dried under nitrogen stream and then reconstituted for injections. Compounds separation and detection was performed by HPLC (Agilent 1290 infinity, San Jose, CA, USA) coupled to a triple quadrupole mass spectrometer (Agilent 6460, San Jose, CA, USA) using an Ascentis® Express column (2.1x150 mm, 2.7 µm particles; Supelco, Bellefonte, PA, USA) during a 28-min gradient. |
| *Amines profiling* | Amino acids and biogenic amines are covered in this platform. Each 25 μL of serum sample was spiked with an internal standard solution and methanol was added. After centrifuging, the supernatant was transferred and dried and then derivatized by AQC reagent for reconstitution. The reaction mixture was injected into the UPLC-MS/MS system which is the ACQUITY ultra-performance liquid chromatography system coupled to a triple quadrupole mass spectrometer (AB SCIEX Qtrap 6500). Chromatographic separation was achieved within a 11-min gradient and followed by positive ion mode detention. |
