# Supplementary material for: Baseline metabolic profiles of early rheumatoid arthritis patients achieving sustained drug-free remission after initiating treat-to-target tocilizumab, methotrexate, or the combination: insights from systems biology
Source: Arthritis Res Ther. 2018 Oct 15;20:230. doi: 10.1186/s13075-018-1729-2 (PMC6235217; doi:10.1186/s13075-018-1729-2)
Supplement: Supplementary file 2 — Overview of the pathway analysis in the (a) tocilizumab plus methotrexate, (b) tocilizumab, and (c) methotrexate strategy arms. The top three most relevant pathways in the tocilizumab plus methotrexate arm were (1) “histidine metabolism,” (2) “sphingolipid metabolism,” and (3) “arachidonic acid metabolism;” in the tocilizumab arm, these were (1) “arachidonic acid metabolism,” (2) “lysine degradation,” and (3) “cysteine and methionine metabolism;” in the methotrexate arm, these were (1) “arginine and proline metabolism,” (2) “histidine metabolism,” and (3) “aminocyl-tRNA biosynthesis.” KEGG Kyoto Encyclopedia of Genes and Genomes, tRNA transfer ribonucleic acid. The colors of the nodes, varying from yellow to red, indicates the level of significance with red being highly significant; the size of the nodes depicts the impact of the pathway with larger nodes illustrating a higher impact. (DOCX 54 kb) [file 13075_2018_1729_MOESM2_ESM.docx]

| **** |
| --- |
| **Additional file 2:** Overview of the pathway analysis in the (**a**) tocilizumab plus methotrexate, (**b**) tocilizumab, and (**c**) methotrexate arm. The top three most relevant pathways in the tocilizumab plus methotrexate arm were (1) “histidine metabolism”, (2) “sphingolipid metabolism”, and (3) “arachidonic acid metabolism”; in the tocilizumab these were (1) “arachidonic acid metabolism”, (2) “lysine degradation”, and (3) “cysteine and methionine metabolism”; in the methotrexate arm these were (1) “arginine and proline metabolism”, (2) “histidine metabolism”, and (3) “aminocyl-tRNA biosynthesis”. KEGG = Kyoto Encyclopedia of Genes and Genomes, tRNA = transfer ribonucleic acid. The colours of the nodes, varying from *yellow* to *red*, indicates the level of significance with *red* being highly significant; the size of the nodes depicts the impact of the pathway with larger nodes illustrating a higher impact. |
